# Patient satisfaction and digital health in primary health care: a scoping review protocol

**DOI:** 10.3389/fpubh.2024.1357688

**Published:** 2024-07-31

**Authors:** Pedro Bezerra Xavier, Ísis de Siqueira Silva, Thaissa Hamana de Macedo Dantas, Rayssa Horácio Lopes, Aguinaldo José de Araújo, Renan Cabral de Figueirêdo, Severina Alice da Costa Uchôa

**Affiliations:** ^1^Center for Health Sciences, Postgraduate Program in Health Sciences, Federal University of Rio Grande do Norte, Natal, Rio Grande do Norte, Brazil; ^2^Center for Health Sciences, Postgraduate program in Collective Health, Federal University of Rio Grande do Norte, Natal, Rio Grande do Norte, Brazil; ^3^School of Health, Federal University of Rio Grande do Norte, Natal, Rio Grande do Norte, Brazil; ^4^Center for Health Sciences, Department of Collective Health, Postgraduate Program in Family Health, Federal University of Rio Grande do Norte, Natal, Rio Grande do Norte, Brazil

**Keywords:** patient satisfaction, telemedicine, primary health care, digital health, patient-centered care, health information technology

## Abstract

**Introduction:**

Using digital health in primary health care (PHC) contributes to reducing costs and travel time, achieving global development goals, improving access, quality and longitudinality of care, and managing health crises. Its evaluation must go beyond the technical-operational aspects to include patient satisfaction, a key element in assessing the quality of care.

**Objective:**

To identify and map patient satisfaction (expectations, desires, cultural values) about the adoption of digital health strategies and assess their impact on the quality of care in PHC.

**Methods:**

The review will follow the recommendations proposed by the Joanna’s Briggs Institute (JBI) manual, the Preferred Reporting Items for Systematic Reviews and Meta-Analyses Extension for Scoping Reviews (PRISMA-ScR) and the methodology proposed by Arksey and O’Malley and Levac et al. and will be conducted in nine stages. The search will be conducted in health studies databases (MEDLINE via PubMed, EMBASE, CINAHL, Web of Science, and BVS), gray literature, and preprint repositories (Google Scholar and MedRxiv). Two reviewers will select the studies, and the third will analyze possible conflicts. The inclusion criteria comprise studies that have been made available in their entirety, whether they are primary studies or short communications, as well as the following materials extracted from the gray literature: preprints, manuals, government documents, books, guidelines, theses and dissertations. Exclusion criteria include literature reviews, abstracts, books, conference archives, letters to the editor, duplicates and opinion articles. Data will be analyzed by content analysis and inferential statistics. This protocol is registered on the Open Science Framework (OSF) under DOI 10.17605/OSF.IO/PUJDB.

**Results:**

The study aims to understand aspects related to the expectations, desires, and cultural values of patients from different countries, as well as the strengths and critical nodes of the use of digital health on the quality of care in PHC.

## 1 Introduction

Digital health is an umbrella term that covers, in addition to telemedicine and Information and Communication Technologies (ICTs), emerging areas of health technology such as artificial intelligence, genomics, and big data (1). ICTs, in turn, are the set of resources used to transmit, store, create, share, or exchange information, such as computers, smartphones, notebooks, e-mail, cameras, and webcams (2).

Using digital tools to answer health questions is not a recent phenomenon. Authors have reported its implementation since the 1960s (3), with more evident growth in the last 30 years (4). During the three decades of its implementation, digital health has made it possible to offer and maintain care for people with mobility difficulties, living in remote or rural areas, access to specialized care even in regions that are difficult to reach, offer and monitor rehabilitation, care for patients in the post-operative recovery phase, and health education (5).

During the COVID-19 pandemic (2020–2023), when distancing measures led to the need to adapt health services at all levels of care (6), digital health stood out as a care strategy that made it possible to maintain health care, minimizing the risks of contagion for professionals and patients (4, 7–9). Even in countries with a long history of implementing digital health, such as Australia (10), the United States (11, 12), Canada (13), and England (14, 15), its use was more restricted to remote or rural regions, as well as hospitals. However, during the COVID-19 pandemic, there has been exponential growth in the adoption of digital health as a care strategy in primary health care (PHC) and as an effective tool in facing the challenges related to epidemiological monitoring of the population, clinical management of COVID-19 (16) and also maintaining support for pre-existing PHC demand, such as people with chronic diseases and pregnant women (7), contributing to the longitudinal care, and reducing the exposure of professionals and patients to unhealthy environments (7, 17–20).

At a time when the world is overcoming one of the biggest pandemics in recent history, achieving Universal Health Coverage (UHC) is one of the goals set by all the world’s nations when they adopted the Sustainable Development Goals (SDGs) (21). Improving health coverage and outcomes requires that professionals have the skills to provide integrated, quality, people-focused care that is available and accessible (21). In this context, primary health care is the most effective and cost-effective way to achieve universal health coverage worldwide. In line with this, digital tools are being incorporated into health care, with the potential to increase access to health services (21).

Although issues related to connectivity, management, and access to ICTs, platform inconsistencies, limitations in performing physical examinations, and difficulties in use by some groups (older people, children, etc.) can negatively impact some processes (22), the implementation of digital health strategies in PHC is a crucial point for reducing financial and time costs related to travel (4, 23), greater access to care in PHC, managing the challenges imposed on health systems, achieving global development goals and improving aspects such as coverage, access and quality (1, 17), also acting as an essential element in treatment adherence and follow-up (24, 25).

The expansion of digital health in the face of the health crisis has highlighted challenges and benefits that need to be assessed and turned into lessons to consolidate improvements in primary health care (26). To this end, digital health in PHC should not only be analyzed from the point of view of logistics and technical-organizational aspects but should also consider patient satisfaction since this is a critical element in the evaluation of the care provided and the search for improvements (6) and PHC should be aligned with the needs, expectations, and values of the population assisted (19). Patient satisfaction is a relevant component of the transition to digital health services, and together with service provision and multisectoral actions it plays a central role in achieving health and well-being (27–29).

Patient satisfaction can be considered a health outcome and, in this study, it will be evaluated based on Donabedian’s model (30, 31), known as “Structure-Process-Outcome.” This assessment reflects patients’ perceptions of the quality of healthcare received and covers several domains, including patient experience (quality of care, kindness and empathy of healthcare professionals, waiting time, ease of communication and response to patient concerns), quality of communication, access to healthcare services (availability of appointments and adequate resources, geographical proximity of services, affordability), satisfaction with treatment and outcomes (respect for patient dignity and privacy, cooperation and coordination of care, patient empowerment and participation), and organizational culture and physical environment (30, 31). In this sense, these domains highlight the importance of considering patient satisfaction as an integral part of providing quality healthcare.

An exploratory search carried out in February 2024 on PubMed/Medline and Google Scholar found some reviews (32–34) assessing patient satisfaction with digital care in PHC. However, no published or registered studies were found (a search was carried out on the Open Science Framework), proposing to evaluate user satisfaction with primary care mediated by digital technologies and aimed at the essential attributes of PHC (First contact service/entry point; Longitudinality; Comprehensiveness or integrality; Coordination of care; Community orientation; Family-centeredness; Cultural competence) (35).

Understanding these aspects from the patients point of view can be crucial to identifying barriers and disparities in primary care through digital health, playing an essential role in planning and implementing the policies and actions needed to adapt strategies and thus achieve satisfactory results and improve the quality of care in PHC. This study aims to base a scoping review protocol to identify and map patient satisfaction (expectations, desires, cultural values) about the adoption of digital health strategies and assess their impact on the quality of care in PHC.

## 2 Conceptual underpinnings

In order to systematize how the questions of interest will be answered, the authors have organized a conceptual theoretical model to represent how patient satisfaction will be assessed in the use of ICTs and their impact on the quality of care in PHC. The ICT is a set of various technological tools and resources to transmit, store, create, share, or exchange information (Personal computers—PCs; Video and photo cameras for computers or webcams; Media for storing and carrying data such as hard disks or hard drives, memory cards, and USB sticks; cell phones, and more advanced ones artificial intelligence, big data, internet, cloud solutions, among others) (1, 36).

User satisfaction will be anchored in Donabedian’s health quality assessment model of structure, process and outcome (30, 31). This was chosen because it is recognized, comprehensive, holistic, flexible and easy to use in the context of PHC in different countries (37, 38). Its systemic vision allows ICTs to be encompassed as an intervention in the health system. The structure indicates the availability and quality of technological resources (electronic health records, telemedicine, among others), the process goes through the way they are applied, and the results include the effects on the effectiveness of digital care and, among these, those perceived by patients – patient satisfaction. Although satisfaction occurs in the professional-patient relational space, this study prioritizes patients’ personal and direct experience of digital primary care.

To verify patient satisfaction with the use of digital health strategies in PHC in the documents included in the final sample, the following qualitative indicators will be considered: satisfaction with treatment and outcomes (respect for patient dignity and privacy; patient empowerment and participation); quality of communication; access to health services (availability of appointments and adequate resources to meet patient needs, geographical proximity of services, affordability); patient experience (quality of care received, including kindness and empathy of health professionals, waiting time, ease of communication and response to patient concerns); cooperation and coordination of care; organizational culture and physical environment.

From a conceptual point of view, there is an interconnection between the use of ICTs, patient satisfaction and essential PHC attributes such as first contact service/entry point; Longitudinality; Comprehensiveness or integrality; Coordination of care; Community orientation; Family-centeredness; Cultural competence (35, 39). As illustrations of the potential of strengthening attributes through the use of ICTs, mobile symptom screening applications can act as an accessible entry point, telemedicine allows continuity of care over time (longitudinality); online platforms are able to integrate services offering a comprehensive approach to healthcare; interoperable electronic health records facilitate effective coordination between health professionals, while social media dedicated to health is able to promote community orientation and applications facilitate coordination of care, and respect and respond effectively to the needs and expectations of patients from different cultural, ethnic, religious and social backgrounds (cultural competence) (40, 41).

In relation to the expected results of the impact of digital primary care, it is considered that satisfied patients are generally associated with better clinical results, adherence to treatment and engagement in healthcare (42). In addition, patient satisfaction can reflect the effectiveness of communication between patients and health professionals, the accessibility of services, respect for the dignity and autonomy of the patient, among other aspects that directly influence the perceived quality of care (43).

With regard to PCC (Population, Concept, and Context), questions are raised about which countries have experience with the use of digital tools in PHC (macro level of study observation) and the types of tools used in health services (meso level). At the micro level of patient satisfaction, the perception of satisfaction will be focused on patients as the study population due to their direct link to health outcomes, such as adherence, health promotion, prevention, treatment and recovery.

## 3 Materials and methods

Scoping reviews seek answers to broad questions, using less restrictive criteria than other types of literature review, and desire, in the inclusion of scientific articles and gray literature, to map the key concepts, types of study, and knowledge gaps in the scope of the established topic, to synthesize the knowledge within it (44). To draw up this protocol, we used the guidelines for scoping reviews contained in the Joanna’s Briggs Institute (JBI) Manual (45), the Preferred Reporting Items for Systematic Reviews and Meta-Analyses Extension for Scoping Reviews (PRISMA-ScR) (44), and the methodology proposed by Arksey and O’Malley (46) and Levac et al. (47). We registered this protocol on the Open Science Framework (OSF) (48).

**Figure 1:**
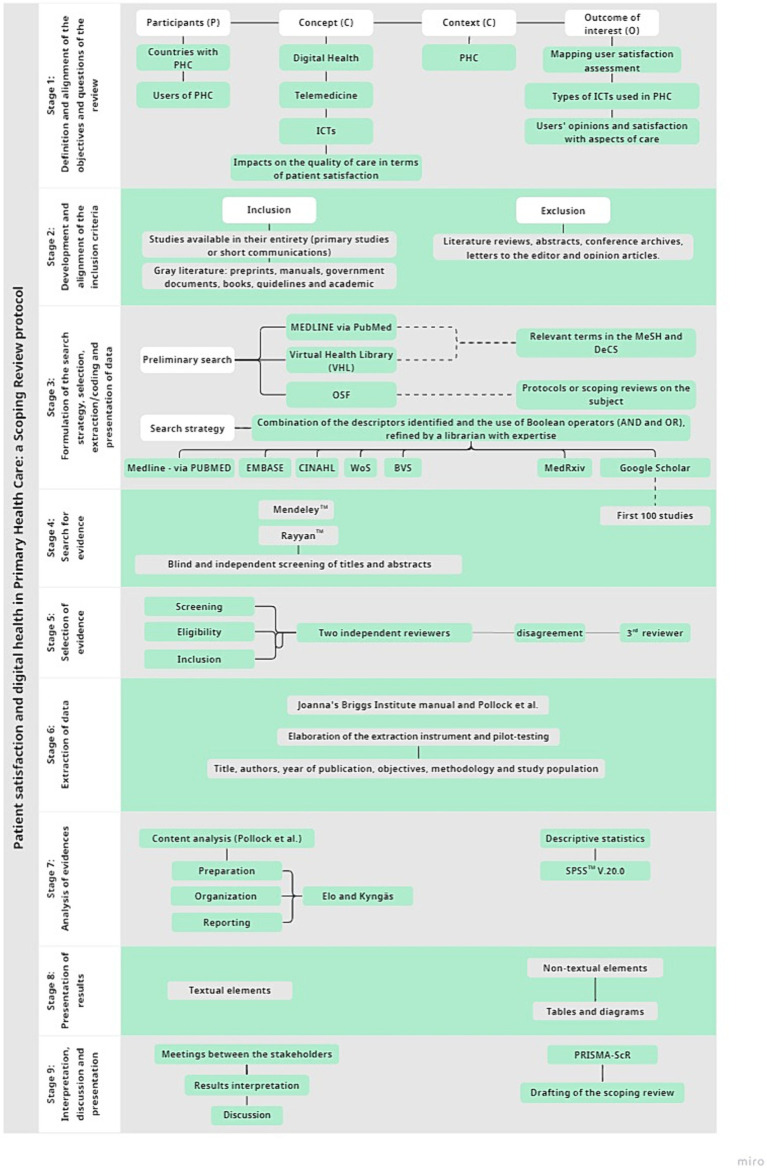
Flowchart illustrating the stages of the study methodology.

The scoping review will be conducted in nine stages (41), namely: (1) Definition and alignment of the objectives and questions of the review; (2) Development and alignment of the inclusion criteria with the objectives of the study; (3) Formulation of the search strategy, selection, extraction/coding and presentation of data; (4) Search for evidence; (5) Selection of evidence; (6) Extraction of data; (7) Analysis of evidence; (8) Presentation of results and (9) Interpretation, discussion and presentation of the implications of the study’s findings. Figure 1 shows the flowchart of the study stages.

### 3.1 Definition and alignment of the objectives and questions of the review

According to the guidelines of Peters et al. (49), after establishing the research objectives, we formulated the questions according to the PCC mnemonic (Population – Concept – Context) plus the outcomes of interest (O). The questions established in consensus by the authors and their basic concepts are detailed in Tables 1, 2, respectively.

**Table 1:** Survey questions.

Question	Participants (P)	Concept (C)	Context (C)	The outcome of interest (O)
1. Which countries have assessed patients’ satisfaction with using digital health strategies in PHC?	Countries with primary health care in the World	Digital health; telemedicine	Primary health care	Geographical mapping of patients’ satisfaction with digital health strategies in PHC.
2. What types of ICTs used in PHC were the subject of evaluating patients’ satisfaction?	Patients of PHC health services	Information and communication technologies (ICTs)	Primary health care	The types of information and communication technologies used in PHC in studies addressing patient satisfaction.
3. What is the health service patients’ perception of adapting digital health strategies to their expectations, desires, cultural values, and about their impact on the quality of care in PHC?	Patients of PHC health services	Impacts on the quality of care in terms of patients satisfaction	Primary health care	Patient satisfaction with digital primary health care.

**Table 2:** Key concepts for the research questions.

Concept	Definition
Digital health (or eHealth)	The umbrella term (telemedicine, telehealth, telecare, among others) covers the use of electronic and mobile technologies (Information and Communication Technology – ICT) (a set of technological resources integrated through hardware, Software, and telecommunications) to support and promote remote clinical healthcare, patient and professional education, public health and healthcare administration (36).
Information and communication technologies	Definition: a set of various technological tools and resources to transmit, store, create, share, or exchange information. Examples of ICTs: Personal computers (PCs); video and photo cameras for computers or webcams; media for storing and carrying data such as hard disks or hard drives, memory cards, and USB sticks; cell phones, and more advanced ones (artificial intelligence, big data, internet, cloud solutions, among others) (1, 36).
Patient satisfaction with health services	Satisfaction with treatment and outcomes (respect for patient dignity and privacy; patient empowerment and participation); quality of communication; access to health services (availability of appointments and adequate resources to meet patient needs, geographical proximity of services, affordability); patient experience (quality of care received, including kindness and empathy of health professionals, waiting time, ease of communication and response to patient concerns); cooperation and coordination of care; organizational culture and physical environment (30, 31).

### 3.2 Development and alignment of the inclusion criteria with the objectives of the study

Inclusion criteria comprise studies that have been made available in their entirety, whether they are primary studies or short communications, as well as the following materials extracted from the gray literature: preprints, manuals, government documents, books, guidelines, theses and dissertations that meet the research questions set out in Table 1.

Exclusion criteria include literature reviews, abstracts, books, conference archives, letters to the editor, duplicates and opinion articles. Documents that do not answer the research questions and do not include the CCP of interest (Table 1) will be excluded. No time or language filters will be applied, and if necessary, we will contact an external translator. A diagram of the study selection process will be available in the final review.

### 3.3 Formulation of the search strategy, selection, extraction/coding, and presentation of data

A preliminary search was carried out on MEDLINE via PubMed and the Virtual Health Library (VHL) to identify terms relevant to the study in the Medical Subject Headings (MeSH) and Descritores em Ciências da Saúde (DeCS). In addition, we conducted an exploratory search on the Open Science Framework (OSF) database to identify possible protocols or scoping reviews on the subject that are being undertaken or completed. Finally, a librarian refined the search strategy based on the combination of the descriptors identified and the use of Boolean operators (AND and OR), with the relevant adjustments for each database. A complete search strategy for MEDLINE via the PubMed database is included in Appendix 1. The final review will explain the detailed strategy for the other bases.

The selection of databases prioritized those that bring together multidisciplinary studies in the health sciences (MEDLINE/PubMed, EMBASE, CINAHL, Web of Science, and VHL), gray literature, and preprint repositories (Google Scholar and MedRxiv).

### 3.4 Search for evidence

Two reviewers will identify the studies in the selected databases. After compiling the identified studies in a reference manager (Mendeley®) and removing duplicates, the resulting material will be attached to the Rayyan® *software* (50) for blind and independent screening of titles and abstracts by the reviewers. For the results of the Google Scholar search, due to the volume of studies identified, the analysis will focus on the first 100 studies, in order of relevance (51).

### 3.5 Selection of evidence

The screening, eligibility within the topic, and inclusion stages will be conducted independently by two reviewers, with a third elected for consultation in the event of disagreement to reach a consensus. Initially, there will be an independent screening of titles and abstracts. In this phase, each of the independent reviewers will check the alignment of the evidence, to identify elements in the titles and abstracts that answer the research question, and the PCC of this study (Table 1), those that do not fit will be excluded. Next, the relevant studies to the review will be extracted and compiled into a new database for further reading of full text and detailed analysis according to the inclusion criteria established. The reference lists of the studies included in the final sample will be consulted to check for publications of interest. The final scoping review will include the reasons for excluding studies.

### 3.6 Extraction of data

The data will be extracted independently by the reviewers using an instrument based on the one proposed by Joanna’s Briggs Institute manual and by Pollock et al. (52), containing information on the characterization of the studies, such as title, authors, year of publication, objectives, methodology and study population; and information relating to the questions of this study: countries that evaluated patient satisfaction with the use of digital health, ICTs used in PHC subjected to the evaluation of patient satisfaction and their satisfaction and perceptions with the use of digital health in PHC. The instrument is included as an Appendix 2 and will be pilot-tested with five studies to assess its suitability for the research needs. If changes to the form are necessary, they will be reported and justified in the final survey, with the presentation of the adjusted document. As in stage 5, the third reviewer will analyze disagreements regarding the extracted content in a consensus meeting.

### 3.7 Analysis of evidence

According to Pollock (52), scoping reviews should not apply advanced analysis methods. That said, the results will be analyzed considering the analysis quantitative variables (year of publication, type of study, methodology, country, ICTs used) will be analyzed using descriptive statistics and presented in absolute and relative frequencies, such as the recurrence of a given topic among the studies analyzed. The SPSS statistical software version 20.0, will be used for this purpose.

For the basic qualitative analysis, the data will be organized according to the inductive approach to qualitative data analysis, seeking coding structures, creating categories and sub-categories, to synthesize the results as recommended by Pollock et al. (44) for scoping reviews, following the three phases proposed by Elo and Kyngäs (45): (1) preparation; (2) organization; and (3) reporting.

### 3.8 Presentation of results

After analyzing and interpreting the data, the review results will be presented in text compiled into topics according to the questions established. Non-textual elements, such as tables and diagrams, will summarize the relevant findings and facilitate communication with the community (53).

### 3.9 Interpretation, discussion, and presentation of the implications of the study’s findings

The data will be analyzed, interpreted, and discussed in holder meetings. After analyzing and interpreting the results, a summary of the main evidence will be sent to potential stakeholders, who will receive an invitation via email to take part in the stakeholder discussion. Stakeholder consultation will be relevant as it helps to overcome communication barriers and achieve consensus (54). Stakeholder participation shapes decision-making and improves policy implementation (55). After the stakeholders submit their comments, two independent experts, one with expertise in technologies and the other in patient satisfaction, external to this research will analyze the stakeholders’ comments to ensure that there is no overvaluation of some findings to the detriment of others, or that some topic has not been adequately discussed.

Possible implications for services and other studies will be pointed out as the data is presented and discussed based on the literature. The drafting of the scoping review will follow the PRISMA-ScR checklist (44), ensuring strict compliance with the criteria. Gaps in the evidence will be highlighted and the conclusions of the review will be used to inform the need for future research.

## 4 Discussion

According to the PRISMA-ScR extension, adopted in this manuscript, it is necessary to have a review protocol and for it to be available for access (44). In the scoping protocol there is a very challenging stage for its implementation: the creation of a data extraction tool (56). A fundamental characteristic of a high quality review is the development of a review protocol, drawn up in advance and defining the main objectives, characteristics and planned analyses of the review, which should be registered prospectively and be publishable in scientific journals (57).

The purpose of the protocol is to clarify the transparency and reproducibility of the review construction process, minimizing research bias and encouraging its use. Its importance and use in the literature is becoming increasingly recognized, providing greater reliability and transparency in scientific research. Furthermore, when planning a scoping review, it is important to consult protocol repositories beforehand in order to find out if there is not already a review under construction (58).

Scoping review protocols are fundamental for formulating and clarifying the scoping review’s research question. The scoping review has made a name for itself worldwide in the area of synthesizing health evidence, organizing and sharing findings that can enrich practices, policies and future research; revealing flaws in already published studies and better understanding how studies are developed in a given field (56). This study will contribute significantly to rigorous evidence synthesis. It represents a key step in carrying out the scoping review, the aim of which is to identify and map patient satisfaction with the adoption of digital health strategies, as well as to assess the impact of these strategies on the quality of care in PHC.

Delving deeper into patient satisfaction in the context of primary care mediated by digital health is relevant, given the expansion in the use of digital health strategies and the need to evaluate the patient experience when using these tools. This research will use Donabedian’s quality model (30, 31), in which patient satisfaction is seen as an important indicator of healthcare quality and can directly influence care processes and health outcomes. High user satisfaction is generally associated with a better care experience, greater adherence to treatment and, potentially, better long-term health outcomes (59). Considered an important dimension of healthcare quality, it should be applied to digital health in general and particularly in PHC.

The interest in PHC services is justified because they focus on individuals and the community, with health promotion, prevention and treatment actions. Their broad domains take into account accessibility, communication with health professionals, experience during treatment and perceived results, among others (59). The implementation of digital health in PHC services can boost trust and the bond between patients and professionals. That said, investigating how patients are accepting technological tools in health contributes to the continuous improvement of the quality of primary health care (17).

One of the strengths of this study is that it presents a comprehensive view of user satisfaction with care mediated by digital technologies. Evaluating the quality of care from the patient’s perspective can provide valuable paths for developing strategies and policies that promote the effective integration of digital health into PHC, with the aim of expanding the positive impacts of primary health care.

A limitation of this study is the large volume of data that will be retrieved from white and gray literature. In order to overcome this challenge, the team has researchers experienced in the development and publication of protocols and scoping reviews, who adopt strategies in the early stages of the evidence selection process, so that the most relevant evidence is mined. The title and abstract reading stage should be carried out with care and will require dedication from the independent reviewers; this is expected to be the longest stage, taking an average of 3 months. Greater dedication to this stage could avoid reading full texts that have no potential for inclusion in this review.

To minimize bias in the presentation of the results, the authors will adopt strategies to ensure transparency in the communication of the results, consult a variety of stakeholders to obtain different perspectives, validate the findings through independent peer review and maintain an impartial position in the interpretation of the results. It is also essential to disclose in detail the stakeholder consultation process, including who was consulted, how their contributions were integrated and how decisions were made to reflect a balanced representation of the findings.

## 5 Conclusion

The protocol was carefully designed to ensure a detailed and reliable analysis, which is essential for recognizing effective practices and identifying areas that require significant improvement. Therefore, this protocol will provide the methodological robustness that will guide a scoping review capable of synthesizing current scientific knowledge. The results elucidated could be valid for the development of a more connected, responsive health system centered on the needs of patients.

The Rayyan program and SPSS will be used for the strategy of organizing and summarizing the results. This procedure will help to increase the processing of the documents that will be retrieved from the literature. The development of a specific extraction form for this study will optimize the time taken to extract the most relevant information.

The inclusion of stakeholders in the analysis of the main evidence will strengthen the sharing of results, and socialization with the interested public, both in the academic environment and with the community, who will, in this sense, be the main affected parties. Changes to this protocol will be duly reported in the final publication, including dates and justifications.

## Author contributions

PX: Writing – original draft, Visualization, Validation, Methodology, Writing – review & editing, Formal analysis. ÍS: Writing – review & editing, Writing – original draft, Visualization, Methodology, Formal analysis, Conceptualization. TD: Writing – review & editing, Writing – original draft, Methodology, Investigation, Formal analysis, Conceptualization. RL: Writing – review & editing, Writing – original draft, Validation, Software, Methodology, Formal analysis, Conceptualization. AA: Writing – review & editing, Writing – original draft, Visualization, Software, Resources, Methodology, Formal analysis. RF: Writing – review & editing, Writing – original draft, Validation, Methodology, Formal analysis. SU: Project administration, Writing – review & editing, Writing – original draft, Visualization, Validation, Supervision, Funding acquisition, Conceptualization.

## References

[1] World Health Organization. WHO guideline: recommendations on digital interventions for health system strengthening. Geneva: World Health Organization (2019). 31162915

[2] World Health Organization. Telemedicine: opportunities and developments in member states: report on the second global survey on eHealth 2009. Geneva: World Health Organization (2010).

[3] Currell R Urquhart C Wainwright P Lewis R. Telemedicine versus face-to-face patient care: effects on professional practice and health care outcomes. Cochrane Database Syst Rev. (2000) 2:CD002098. doi: 10.1002/14651858.CD002098 10796678

[4] Kruse CS Krowski N Rodriguez B Tran L Vela J Brooks M. Telehealth and patient satisfaction: a systematic review and narrative analysis. BMJ Open. (2017) 7:e016242. doi: 10.1136/bmjopen-2017-016242, PMID: 28775188 PMC5629741

[5] Flodgren G Rachas A Farmer AJ Inzitari M Shepperd S. Interactive telemedicine: effects on professional practice and health care outcomes. Cochrane Database Syst Rev. (2015) 2015:CD002098. doi: 10.1002/14651858.CD002098.pub2, PMID: 26343551 PMC6473731

[6] Lukas H Xu C Yu Y Gao W. Emerging telemedicine tools for remote COVID-19 diagnosis, monitoring, and management. ACS Nano. (2020) 14:16180–93. doi: 10.1021/acsnano.0c08494, PMID: 33314910

[7] Matenge S Sturgiss E Desborough J Hall Dykgraaf S Dut G Kidd M. Ensuring the continuation of routine primary care during the COVID-19 pandemic: a review of the international literature. Fam Pract. (2022) 39:747–61. doi: 10.1093/fampra/cmab115, PMID: 34611708 PMC8515263

[8] Thirunavukkarasu A Alotaibi NH Al-Hazmi AH Alenzi MJ Alshaalan ZM Alruwaili MG . Patients’ perceptions and satisfaction with the outpatient telemedicine clinics during COVID-19 era in Saudi Arabia: a cross-sectional study. Healthcare (Basel). (2021) 9:1739. doi: 10.3390/healthcare9121739 34946465 PMC8701957

[9] Monaghesh E Hajizadeh A. The role of telehealth during COVID-19 outbreak: a systematic review based on current evidence. BMC Public Health. (2020) 20:1193. doi: 10.1186/s12889-020-09301-4, PMID: 32738884 PMC7395209

[10] Toll K Spark L Neo B Norman R Elliott S Wells L . Consumer preferences, experiences, and attitudes towards telehealth: qualitative evidence from Australia. PLoS One. (2022) 17:e0273935. doi: 10.1371/journal.pone.0273935, PMID: 36044536 PMC9432716

[11] DeMonte CM DeMonte WD Thorn BE. Future implications of eHealth interventions for chronic pain management in underserved populations. Pain Manage. (2015) 5:207–14. doi: 10.2217/pmt.15.9, PMID: 25971644

[12] Chesser A Burke A Reyes J Rohrberg T. Navigating the digital divide: a systematic review of eHealth literacy in underserved populations in the United States. Inform Health Soc Care. (2016) 41:1–19. doi: 10.3109/17538157.2014.948171, PMID: 25710808

[13] Warmington K Flewelling C Kennedy CA Shupak R Papachristos A Jones C . Telemedicine delivery of patient education in remote Ontario communities: feasibility of an advanced clinician practitioner in arthritis care (ACPAC)-led inflammatory arthritis education program. Open Access Rheumatol. (2017) 9:11–9. doi: 10.2147/OARRR.S122015, PMID: 28280400 PMC5338940

[14] Buckingham SA Sein K Anil K Demain S Gunn H Jones RB . Telerehabilitation for physical disabilities and movement impairment: a service evaluation in south West England. J Eval Clin Pract. (2022) 28:1084–95. doi: 10.1111/jep.13689, PMID: 35437833 PMC9790516

[15] Winward S Patel T Al-Saffar M Noble M. The effect of 24/7, digital-first, NHS primary care on acute hospital spending: retrospective observational analysis. J Med Internet Res. (2021) 23:e24917. doi: 10.2196/24917, PMID: 34292160 PMC8367118

[16] Koonin LM Hoots B Tsang CA Leroy Z Farris K Jolly T . Trends in the use of telehealth during the emergence of the COVID-19 pandemic-United States, January–March 2020. MMWR Morb Mortal Wkly Rep. (2020) 69:1595–9. doi: 10.15585/mmwr.mm6943a3, PMID: 33119561 PMC7641006

[17] Silva CRDV Lopes RH de Goes BO Martiniano CS Fuentealba-Torres M Arcêncio RA . Digital health opportunities to improve primary health care in the context of COVID-19: scoping review. JMIR Hum Factors. (2022) 9:e35380. doi: 10.2196/35380, PMID: 35319466 PMC9159467

[18] Judson TJ Odisho AY Neinstein AB Chao J Williams A Miller C . Rapid design and implementation of an integrated patient self-triage and self-scheduling tool for COVID-19. J Am Med Inform Assoc. (2020) 27:860–6. doi: 10.1093/jamia/ocaa051, PMID: 32267928 PMC7184478

[19] Drerup B Espenschied J Wiedemer J Hamilton L. Reduced no-show rates and sustained patient satisfaction of telehealth during the COVID-19 pandemic. Telemed J E Health. (2021) 27:1409–15. doi: 10.1089/tmj.2021.0002, PMID: 33661708

[20] Verhoeven V Tsakitzidis G Philips H Van Royen P. Impact of the COVID-19 pandemic on the core functions of primary care: will the cure be worse than the disease? A qualitative interview study in Flemish GPs. BMJ Open. (2020) 10:e039674. doi: 10.1136/bmjopen-2020-039674, PMID: 32554730 PMC7306272

[21] World Health Organization. WHO Cobertura Universal de Saúde (CUS). Geneva: World Health Organization (2023).

[22] Combi C Pozzani G Pozzi G. Telemedicine for developing countries. A survey and some design issues. Appl Clin Inform. (2016) 7:1025–50. doi: 10.4338/ACI-2016-06-R-0089 27803948 PMC5228142

[23] Stoumpos AI Kitsios F Talias MA. Digital transformation in healthcare: technology acceptance and its applications. Int J Environ Res Public Health. (2023) 20:3407. doi: 10.3390/ijerph20043407, PMID: 36834105 PMC9963556

[24] Jabeen R Salman MJ Qazi I. Evidence of mobile health integration into primary health care systems for better maternal mental health in LMICs during COVID-19 pandemic – review. J Pak Med Assoc. (2023) 73:125–8. doi: 10.47391/JPMA.5155 36842020

[25] Vicente MA Fernández C Guilabert M Carrillo I Martín-Delgado J Mira JJ . Patient engagement using telemedicine in primary care during COVID-19 pandemic: a trial study. Int J Environ Res Public Health. (2022) 19:14682. doi: 10.3390/ijerph192214682, PMID: 36429402 PMC9690471

[26] Neves AL Burgers J. Digital technologies in primary care: implications for patient care and future research. Eur J Gen Pract. (2022) 28:203–8. doi: 10.1080/13814788.2022.2052041, PMID: 35815445 PMC9278419

[27] Chotchoungchatchai S Marshall AI Witthayapipopsakul W Panichkriangkrai W Patcharanarumol W Tangcharoensathien V. Primary health care and sustainable development goals. Bull World Health Organ. (2020) 98:792–800. doi: 10.2471/BLT.19.245613 33177776 PMC7607463

[28] De Rosis S Barsanti S. Patient satisfaction, e-health and the evolution of the patient-general practitioner relationship: evidence from an Italian survey. Health Policy. (2016) 120:1279–92. doi: 10.1016/j.healthpol.2016.09.012, PMID: 27836231

[29] Ministério da Saúde (Brasil). Satisfação do Cliente Agência Nacional de Saúde Suplementar. Brasília (BR): Ministério da Saúde (2012).

[30] Donabedian A. Twenty years of research on the quality of medical care: 1964-1984. Eval Health Prof. (1985) 8:243–65. doi: 10.1177/016327878500800301 10301003

[31] Donabedian A. The quality of care. How can it be assessed? JAMA. (1988) 260:1743–8. doi: 10.1001/jama.1988.03410120089033 3045356

[32] Viitanen J Valkonen P Savolainen K Karisalmi N Hölsä S Kujala S. Patient experience from an eHealth perspective: a scoping review of approaches and recent trends. Yearb Med Inform. (2022) 31:136–45. doi: 10.1055/s-0042-1742515, PMID: 36463871 PMC9719751

[33] Alashek WA Ali SA. Satisfaction with telemedicine use during COVID-19 pandemic in the UK: a systematic review. Libyan J Med. (2024) 19:2301829. doi: 10.1080/19932820.2024.2301829, PMID: 38197179 PMC10783830

[34] Du Y Gu Y. The development of evaluation scale of the patient satisfaction with telemedicine: a systematic review. BMC Med Inform Decis Mak. (2024) 24:31. doi: 10.1186/s12911-024-02436-z, PMID: 38303031 PMC10832124

[35] Starfield B. Primary care: balancing health needs, services, and technology, vol. ix. Rev. ed. New York: Oxford University Press (1998). 438 p.

[36] World Health Organization. Global diffusion of eHealth: making universal health coverage achievable: report of the third global survey on eHealth. Geneva, Switzerland: World Health Organization (2016).

[37] Reis EJFB Santos FP Campos FE Acúrcio FA Leite MTT Leite MLC . Avaliação da qualidade dos serviços de saúde: notas bibliográficas. Cad Saude Publica. (1990) 6:50–61. doi: 10.1590/S0102-311X1990000100006

[38] Starfield B. Atenção primária: equil Ìbrio entre necessidades de saúde, serviços e tecnologia/Bárbara Starfield. Brasília: UNESCO, Ministério da Saúde (2002). 726 p.

[39] Bender JD Facchini LA Lapão LMV Tomasi E Thumé E. O uso de Tecnologias de Informação e Comunicação em Saúde na Atenção Primária à Saúde no Brasil, de 2014 a 2018. Ciênc Saúde Coletiva. (2024) 29:e19882022. doi: 10.1590/1413-81232024291.19882022, PMID: 38198338

[40] Ming LC Untong N Aliudin NA Osili N Kifli N Tan CS . Mobile health apps on COVID-19 launched in the early days of the pandemic: content analysis and review. JMIR Mhealth Uhealth. (2020) 8:e19796. doi: 10.2196/19796, PMID: 32609622 PMC7505686

[41] Maaß L Freye M Pan CC Dassow HH Niess J Jahnel T. The definitions of health apps and medical apps from the perspective of public health and law: qualitative analysis of an interdisciplinary literature overview. JMIR Mhealth Uhealth. (2022) 10:e37980. doi: 10.2196/37980, PMID: 36315221 PMC9664324

[42] Durmuş A Akbolat M. The impact of patient satisfaction on patient commitment and the mediating role of patient trust. J Patient Exp. (2020) 7:1642–7. doi: 10.1177/2374373520967807, PMID: 33457625 PMC7786692

[43] Villar VCFL Martins M Rabello ET. Qualidade do cuidado e segurança do paciente: o papel dos pacientes e familiares. Saúde em Debate. (2024) 46:1174–86. doi: 10.1590/0103-1104202213516

[44] Tricco AC Lachance CC Rios P Darvesh N Antony J Radhakrishnan A . Global evidence of gender inequity in academic health research: a living scoping review protocol. JBI Evid Synth. (2020) 18:2181–93. doi: 10.11124/JBIES-20-00078, PMID: 32925395

[45] Aromataris E Munn Z eds. JBI manual for evidence synthesis. Adelaide: JBI (2020).

[46] Arksey H O'Malley L. Scoping studies: towards a methodological framework. Int J Soc Res Methodol. (2005) 8:19–32. doi: 10.1080/1364557032000119616

[47] Levac D Colquhoun H O'Brien KK. Scoping studies: advancing the methodology. Implement Sci. (2010) 5:69. doi: 10.1186/1748-5908-5-69, PMID: 20854677 PMC2954944

[48] Xavier PB Silva ÍdS Dantas THdM Lopes RH Araújo AJd Figueiredo RCd . Patient satisfaction and digital health in primary health care: a scoping review protocol.. Open Science Framework (OSF). (2024). doi: 10.17605/OSF.IO/PUJDB PMC11322341 39145169

[49] MDJ P Godfrey C McInerney P Baldini Soares C Khalil H Parker D. Chapter 11: scoping reviews In: Aromataris E Munn Z, editors. JBI manual for evidence synthesis. Adelaide: JBI (2020)

[50] Ouzzani M Hammady H Fedorowicz Z Elmagarmid A. Rayyan-a web and mobile app for systematic reviews. Syst Rev. (2016) 5:210. doi: 10.1186/s13643-016-0384-4, PMID: 27919275 PMC5139140

[51] Godin K Stapleton J Kirkpatrick SI Hanning RM Leatherdale ST. Applying systematic review search methods to the grey literature: a case study examining guidelines for school-based breakfast programs in Canada. Syst Rev. (2015) 4:138. doi: 10.1186/s13643-015-0125-0, PMID: 26494010 PMC4619264

[52] Pollock D Peters MDJ Khalil H McInerney P Alexander L Tricco AC . Recommendations for the extraction, analysis, and presentation of results in scoping reviews. JBI Evid Synth. (2023) 21:520–32. doi: 10.11124/JBIES-22-00123 36081365

[53] Elo S Kyngäs H. The qualitative content analysis process. J Adv Nurs. (2008) 62:107–15. doi: 10.1111/j.1365-2648.2007.04569.x 18352969

[54] Zikargae MH Woldearegay AG Skjerdal T. Assessing the roles of stakeholders in community projects on environmental security and livelihood of impoverished rural society: a nongovernmental organization implementation strategy in focus. Heliyon. (2022) 8:e10987. doi: 10.1016/j.heliyon.2022.e10987, PMID: 36276717 PMC9582698

[55] Berry LH Koski J Verkuijl C Strambo C Piggot G. Making space: how public participation shapes environmental decision. Stockholm: Stockholm Environment Institute (2019).

[56] Cordeiro L Soares CB. Revisão de escopo: potencialidades para a síntese de metodologias utilizadas em pesquisa primária qualitativa. BIS. (2019) 20:37–43. doi: 10.52753/bis.2019.v20.34471

[57] Mattos SM Cestari VRF Moreira TMM. Protocolo de revisão de escopo: aperfeiçoamento do guia PRISMAScR. Rev Enferm UFPI. (2023) 12:e3062. doi: 10.26694/reufpi.v12i1.3062

[58] Moraes EB. Review protocols [editorial]. Online Braz J Nurs. (2022) 21:e20226585. doi: 10.17665/1676-4285.20226585

[59] World Health Organization. Atenção primária à saúde. Available at: https://www.paho.org/pt/topicos/atencao-primaria-saude

