# Supplementary material for: Patient satisfaction and digital health in primary health care: a scoping review protocol
Source: Front Public Health. 2024 Jul 31;12:1357688. doi: 10.3389/fpubh.2024.1357688 (PMC11322341; doi:10.3389/fpubh.2024.1357688)
Supplement: Supplementary file 1 [file Table_1.docx]

**Appendix 1 –** Database Search Strategy

According to the strategy below, we will search Medline via the PubMed database using Boolean operators AND and OR. We will select title/abstract and MeSH term fields in the advanced search builder.

Complete strategy for the search in Medline via PubMed.

| **Search** | **Terms** | **Retrieved studies**  (October 05, 2023) |
| --- | --- | --- |
| #1 | Search: ("Consumer Behavior"OR "Patient Satisfaction" OR "Quality of Health Care"[MeSH Terms]) OR ("Behavior, Consumer"[Title/Abstract] OR "Behaviors, Consumer"[Title/Abstract] OR "Consumer Behaviors"[Title/Abstract] OR "Consumer Preference"[Title/Abstract] OR "Consumer Preferences"[Title/Abstract] OR "Preference, Consumer"[Title/Abstract] OR "Preferences, Consumer"[Title/Abstract] OR "Consumer Satisfaction"[Title/Abstract] OR "Satisfaction, Consumer"[Title/Abstract] OR "Consumer Behavior"[Title/Abstract] OR "consumer attitude"[Title/Abstract] OR "consumer behavior"[Title/Abstract] OR "satisfaction"[Title/Abstract]) | 8,265,067 |
| #2 | Search: "Quality of Health Care"[Title/Abstract] OR "Care Quality"[Title/Abstract] OR "Health Care Quality"[Title/Abstract] OR "Healthcare Quality"[Title/Abstract] OR "Quality of Care"[Title/Abstract] | 62,272 |
| #3 | Search: ("eHealth Strategies" OR "Information Technology" OR "Telemedicine"[MeSH Terms]) OR (eHeath[Title/Abstract] OR "Digital Health Strategies"[Title/Abstract] OR "Digital Health Strategy["[Title/Abstract] OR TelemedicineOR "Connected Health"[Title/Abstract] OR "Digital Health"[Title/Abstract] OR "Health 2.0"[Title/Abstract] OR "Health Tele-Services"[Title/Abstract] OR "Health Teleservices"[Title/Abstract] OR "Health, Mobile"[Title/Abstract] OR "Medicine 2.0"[Title/Abstract] OR mHealth[Title/Abstract] OR "mHealth Alliance"[Title/Abstract] OR "Mobile Health"[Title/Abstract] OR Telecare[Title/Abstract] OR Telecure[Title/Abstract] OR Telehealth[Title/Abstract] OR "Teleservices in the Health Sector"[Title/Abstract] OR u-Health[Title/Abstract] OR "Health, Mobile"[Title/Abstract] OR "Information Technologies"[Title/Abstract] OR "Technology, Information"[Title/Abstract] OR "Health ICT"[Title/Abstract] OR ICT[Title/Abstract] OR "ICT in Health"[Title/Abstract] OR "ICT Use in Health"[Title/Abstract] OR Information[Title/Abstract] OR "Communication Technologies"[Title/Abstract] OR "Communication Technology"[Title/Abstract] OR "Information Technologies"[Title/Abstract] OR "Technology, Information"[Title/Abstract] OR "information technology"[Title/Abstract]) | 1,663,326 |
| #4 | Search: ("Primary Health Care"[MeSH Terms]) OR ("Primary Health Care"[Title/Abstract] OR "Care, Primary"[Title/Abstract] OR "Care, Primary Health"[Title/Abstract] OR "Health Care, Primary"[Title/Abstract] OR Healthcare, Primary"[Title/Abstract] OR "Primary Care"[Title/Abstract] OR "Primary Healthcare"[Title/Abstract]) | 192,677 |
|  | # 1 AND #2 AND #3 AND #4  Search: (((("Consumer Behavior"OR "Patient Satisfaction" OR "Quality of Health Care"[MeSH Terms]) OR ("Behavior, Consumer"[Title/Abstract] OR "Behaviors, Consumer"[Title/Abstract] OR "Consumer Behaviors"[Title/Abstract] OR "Consumer Preference"[Title/Abstract] OR "Consumer Preferences"[Title/Abstract] OR "Preference, Consumer"[Title/Abstract] OR "Preferences, Consumer"[Title/Abstract] OR "Consumer Satisfaction"[Title/Abstract] OR "Satisfaction, Consumer"[Title/Abstract] OR "Consumer Behavior"[Title/Abstract] OR "consumer attitude"[Title/Abstract] OR "consumer behavior"[Title/Abstract] OR "satisfaction"[Title/Abstract])) AND ("Quality of Health Care"[Title/Abstract] OR "Care Quality"[Title/Abstract] OR "Health Care Quality"[Title/Abstract] OR "Healthcare Quality"[Title/Abstract] OR "Quality of Care"[Title/Abstract])) AND (("eHealth Strategies" OR "Information Technology" OR "Telemedicine"[MeSH Terms]) OR (eHeath[Title/Abstract] OR "Digital Health Strategies"[Title/Abstract] OR "Digital Health Strategy["[Title/Abstract] OR TelemedicineOR "Connected Health"[Title/Abstract] OR "Digital Health"[Title/Abstract] OR "Health 2.0"[Title/Abstract] OR "Health Tele-Services"[Title/Abstract] OR "Health Teleservices"[Title/Abstract] OR "Health, Mobile"[Title/Abstract] OR "Medicine 2.0"[Title/Abstract] OR mHealth[Title/Abstract] OR "mHealth Alliance"[Title/Abstract] OR "Mobile Health"[Title/Abstract] OR Telecare[Title/Abstract] OR Telecure[Title/Abstract] OR Telehealth[Title/Abstract] OR "Teleservices in the Health Sector"[Title/Abstract] OR u-Health[Title/Abstract] OR "Health, Mobile"[Title/Abstract] OR "Information Technologies"[Title/Abstract] OR "Technology, Information"[Title/Abstract] OR "Health ICT"[Title/Abstract] OR ICT[Title/Abstract] OR "ICT in Health"[Title/Abstract] OR "ICT Use in Health"[Title/Abstract] OR Information[Title/Abstract] OR "Communication Technologies"[Title/Abstract] OR "Communication Technology"[Title/Abstract] OR "Information Technologies"[Title/Abstract] OR "Technology, Information"[Title/Abstract] OR "information technology"[Title/Abstract]))) AND (("Primary Health Care"[MeSH Terms]) OR ("Primary Health Care"[Title/Abstract] OR "Care, Primary"[Title/Abstract] OR "Care, Primary Health"[Title/Abstract] OR "Health Care, Primary"[Title/Abstract] OR Healthcare, Primary"[Title/Abstract] OR "Primary Care"[Title/Abstract] OR "Primary Healthcare"[Title/Abstract])) | 1,165 |
