# Supplementary material for: Patient satisfaction and digital health in primary health care: a scoping review protocol
Source: Front Public Health. 2024 Jul 31;12:1357688. doi: 10.3389/fpubh.2024.1357688 (PMC11322341; doi:10.3389/fpubh.2024.1357688)
Supplement: Supplementary file 2 [file Table_2.docx]

| **Study characterization** | |
| --- | --- |
| Title |  |
| Authors |  |
| Year of publication |  |
| Objective |  |
| Type of Study |  |
| Methodology |  |
| Study population |  |
| **User satisfaction with telemedicine in PHC** | |
| Country in which user satisfaction with digital health in PHC was assessed. |  |
| What are the ICTs used in telemedicine in PHC evaluated in the study? |  |
| Patient's perception of the adaptation of digital health strategies to their expectations, desires, and cultural values |  |
| Patient's perception of the impact of digital health on the quality of care in PHC |  |

**Appendix 2 - Data extraction form for included studies**
